# Algodystrophy and pregnancy

**DOI:** 10.11604/pamj.2017.26.117.11797

**Published:** 2017-03-02

**Authors:** Zeineb Alaya, Walid Osman

**Affiliations:** 1 Department of Rheumatology, Farhat Hached Hospital, Faculty of Medicine of Sousse, Sousse, Tunisia; 2 Department of Orthopaedics, Sahloul Hospital, Faculty of Medicine of Sousse, Sousse, Tunisia

**Keywords:** Algodystrophy, pregnancy, MRI

## Image in medicine

The algodystrophy is a regional pain syndrome, characterized by complex neurovascular abnormalities. Pregnancy appears through mechanical factors promote dystrophy of the lower limbs. We report the case of a 31 year old woman, primipara, who presented in the third quarter of pregnancy (35 weeks gestation) mechanical right groin pain with functional impairment. She has consulted in postpartum (4 days after birth). The review noted a limp, functional impairment with a visual analogue scale (VAS) pain 70%. Standard radiographs postpartum showed speckled locoregional demineralization of the right hip. MRI confirmed the diagnosis of right hip algodystrophy (A and B). The patient took analgesic treatment with a discharge and rest for 3 months. She has not received bisphosphonates because she breastfeeding. Disease duration was 24 weeks. The evolution was marked by the persistence of residual pain (VAS 20%) without sequelae. Algodystrophy during pregnancy is rare and is often misunderstood. The analysis of our results compared to literature, allows to identify the main characteristics of this variety of reflex sympathetic dystrophy: gradual onset in the second or third trimester, location in the hip alone or associated with other locations. MRI is currently the modality of choice for the early and differential diagnosis. The course is generally favorable. The safety of bisphosphonates during pregnancy and lactation remains to be demonstrated.

**Figure 1:**
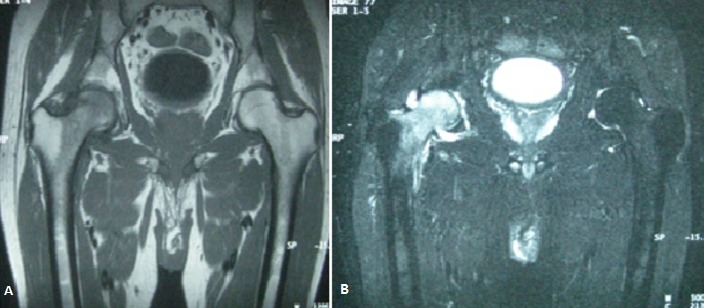
(A) MRI of the hips: diffused medullary edema of the neck and right femoral head in hyposignal T1 and hypersignal STIR with discreet intra-articular effusion blade; (B) suggestive of right hip algodystrophy

